# Implementing a logbook on entrustable professional activities in the final year of undergraduate medical education in Germany – a multicentric pilot study

**DOI:** 10.3205/zma001277

**Published:** 2019-11-15

**Authors:** Kristina Schick, Alexander Eissner, Marjo Wijnen-Meijer, Jonas Johannink, Bert Huenges, Maren Ehrhardt, Martina Kadmon, Pascal O. Berberat, Thomas Rotthoff

**Affiliations:** 1 Technical University of Munich, School of Medicine, Medical Education Center, Munich, Germany; 2 Heinrich Heine University Düsseldorf, Medical Faculty, Dean's Office, Düsseldorf, Germany; 3 Eberhard Karls University of Tübingen, University Department of General, Visceral and Transplant Surgery, Tübingen, Germany; 4 RUB, Faculty of Medicine, Institute of General Practice and Family Medicine, Bochum, Germany; 5 Hamburg University Medical School, Department of General Practice/Primary Care, Hamburg, Germany; 6 Augsburg University, Faculty of Medicine, Dean, Augsburg, Germany; 7 Augsburg University, Faculty of Medicine, Department for Medical Education and Educational Research, Augsburg, Germany

**Keywords:** entrustable professional activities, clinical electives, undergraduate medical education

## Abstract

**Objectives: **The final year of undergraduate medical education (practical year) should foster the transition from undergraduate medical education to graduate medical education. Medical students in the practical year should be able to assume professional tasks, and supervisors should assign these tasks to them. In this pilot study, a curriculum based on the concept of entrustable professional activities (EPAs) was implemented and evaluated in the disciplines of internal medicine, surgery and general practice at four university hospitals.

**Methods:** *n*=37 medical students and *n*=17 supervising physicians at four German university hospitals participated in the implementation study for one trimester. For evaluation purposes, we conducted focus group discussions and telephone interviews and analyzed them following qualitative content analysis.

**Results: **We identified five different aspects as important for implementing the EPA curriculum in undergraduate medical education in the German context:

1. Implementation process of the EPA curriculum and required resources,
2. Entrustment process,
3. Feedback sessions with supervisors,
4. Students’ and supervisors’ role perception
5. Overall impact of EPAs on training conditions in the practical year.

**Conclusion: **The study presents a practical implementation of the EPA curriculum in Germany’s undergraduate medical education. Besides the need for time and resources, the concept shows good feasibility and fosters a competence-oriented undergraduate medical education in the practical year.

## Zusammenfassung

**Hintergrund:** Das letzte Jahr der medizinischen Ausbildung (Praktisches Jahr) soll den Übergang zwischen dem Medizinstudium und der ärztlichen Weiterbildung fördern. Medizinstudierende im Praktischen Jahr sollten befähigt werden, professionelle Aufgaben zu übernehmen. Diese sollten ihnen von Supervisor/innen auch übertragen werden. In dieser Pilotstudie wird ein Curriculum-basiertes Konzept zu anvertraubaren professionellen Tätigkeiten (APT) eingeführt und an vier Universitätskliniken für die Fachbereiche Innere Medizin, Chirurgie und Allgemeinmedizin evaluiert.

**Methode:** *N*=37 Medizinstudierende und *N*=17 supervidierende Ärzt/Ärztinnen an vier deutschen Universitätskliniken nahmen an der Implementierungsstudie für ein Tertial teil. Zur Evaluationszwecken führten wir Fokusgruppen und Telefoninterviews durch und analysierten dessen Transkripte nach einem qualitativ inhaltsanalytischen Ansatz.

**Ergebnisse: **Wir konnten fünf Aspekte identifizieren, die sich als wichtig für die Implementierung eines APT-Curriculums im Medizinstudium im deutschsprachigen Kontext herausgestellt haben:

1. Der Implementationsprozess des APT-Curriculums und die dafür benötigten Ressourcen,
2. Prozess des Anvertrauens,
3. Feedbackgespräche mit Supervisor/innen,
4. Rollenwahrnehmung der Medizinstudierenden und der Supervisor/innen,
5. Gesamteinfluss der APTs auf die Trainingsbedingungen im Praktischen Jahr.

**Schlussfolgerung: **Die Studie zeigt eine praktische Implementierung des APT-Curriculums im deutschsprachigen Medizinstudium. Neben dem Bedarf an Zeit und Ressourcen, zeigt das Konzept gute Durchführbarkeit und fördert eine kompetenzorientierte medizinische Ausbildung im Praktischen Jahr.

## 1. Introduction

In their final year of medical school in Germany, students work full time in clinical departments or outpatient clinics under the supervision and responsibility of instructing physicians to deepen and extend their medical knowledge, skills and abilities according to the students’ level of expertise (§ 3 Abs. 4 ÄApprO 2002 [https://www.gesetze-im-internet.de/_appro_2002/__3.html]).

This final year, also known as the practical year with clinical electives, is the last opportunity for students to obtain clinical practice in the protected environment of undergraduate medical education. After the final medical exam, young physicians have to perform, from one day to another, clinical tasks and activities independently and often without direct supervision. They have to integrate all professional roles to carry out ethical and patient-centered medical care according to their educational level.

Therefore, it is reasonable to hold medical students increasingly responsible during their clinical electives in their last year in medical school [1]. A stepwise entrustment of professional activities to trainees is currently being widely discussed and already partly implemented in several countries in under- and postgraduate medical education based on the concept of entrustable professional activities (EPAs) (Netherlands [2], USA [3] and Switzerland [4]). An EPA is defined as a unit of professional practice that can be entrusted to a trainee [5]. It was introduced at the beginning of this century. The entrustment levels go from direct supervision to full entrustment without supervision as soon as the student demonstrates the necessary competence to execute the particular activity unsupervised [5], [6]. Independent of the size of the EPA, ten Cate defined five different levels [5]:

- level 1 – the trainee is not allowed to perform the task (he/she is completely incompetent or too incompetent),
- level 2 – the trainee is allowed to perform the task under direct supervision or together with the supervisor,
- level 3 – the trainee is allowed to perform the task under indirect supervision (the supervisor is in the ward or available via telephone),
- level 4 – the trainee is allowed to perform the task without supervision and
- level 5 – the trainee is allowed to supervise another trainee or undergraduate student.

Today, “ad hoc” entrustment decisions usually take place where supervising doctors entrust tasks to medical students when they assume that the students are sufficiently able and competent to perform the tasks in an appropriate way, even up to entrustment level 3 described above [5], [7]. However, this process is often rather implicit, unstructured and hardly transparent. A summative entrustment decision is similar to a certification [5]; the physician decides to entrust a given task from that moment on relating to the demonstrated competencies and skills of the trainee.

Based on this background, a working group at the German Society of Medical Schools (“Medizinischer Fakultätentag” [MFT]) developed an EPA curriculum for students in their final year of study for the disciplines of internal medicine, surgery and general practice [8]. Internal medicine and surgery are mandatory electives in the final year, whereas general practice is an exemplary optional elective. This EPA curriculum is based on AMEE Guide No. 99 [5] and Kadmon et al.’s [9] concept. The concept defines coarse-grained EPAs by enumerating the required knowledge, skills and attitudes for each EPA, as well as the supervision scale with the EPA levels. Coarse-grained EPAs define overarching professional tasks – which include different smaller skills and abilities, such as inpatient admission. Besides history taking, requesting test results, assigning a patient´s room to a student and so on are needed to ensure successful and careful patient management. Also, examples of corresponding formative assessments (i.e., Mini-Cex) are provided to support an entrustment decision.

This pilot study will answer the question if the new defined coarse-grained EPAs are feasible and acceptable for the daily clinical teaching routine and which prerequisites or interventions are necessary for their successful implementation.

## 2. The pilot project

The newly developed EPA curriculum was pilot tested during a trimester of the clinical electives of undergraduate medical education (for further information about the EPA curriculum, see Berberat et al. [8], same issue). Sixty-two medical students and 26 supervising physicians in surgery, internal medicine and general practice participated in the pilot study at the university hospitals and at their academic training practice for general medicine at the University of Düsseldorf, the University of Tübingen, the University of Bochum and the Technical University of Munich. Assistant doctors, specialists and senior physicians acted as supervisors.

The medical schools designed their introduction differently. In Düsseldorf, the supervisors were prepared in individual training sessions and the students at the beginning of their rotations. In Munich, Bochum and Tübingen, two introductory sessions were conducted: one for the supervisors and one for the students. The content and amount of the introductory sessions also had different thematic priorities. In Munich, Bochum and Tübingen, the EPA concept, in general, and the newly developed EPA curriculum, in particular, were introduced in these sessions. The purpose and the structure of the EPA were explained and the planned procedure was presented. Two reflection tasks about how they would justify the entrustment decision and how they would use the different entrustment levels were carried out. Furthermore, in Düsseldorf, the tasks focused on reflecting on the goals of the final year and the importance of assuming responsibility. The training of students in the wards was conducted according to the new EPA curriculum and its principles described by Berberat et al. [8]. The supervisor and the students were given a schedule with the dates of feedback sessions and Mini-Cex (see table 1 (Tab. 1)). The project coordinators (AE, BH, JJ and KS) reminded the students and the physicians to hold feedback talks according to the schedule. They coached the medical supervisors on project tasks and sought feedback on the implementation processes in the wards.

For evaluation purposes, we conducted five focus group discussions with a total of *n*=29 (female: 69.0%) students at three university hospitals and telephone interviews with *n*=8 (female: 37.5%) students at the Technical University of Munich University Hospital. The telephone interviews were conducted at the Technical University of Munich as an alternative to the focus group discussions due to organizational reasons. The same questions were used for both approaches. Additionally, *n*=17 supervising physicians were interviewed in four focus group discussions at four university hospitals (*n*=6 general practitioners, *n*=8 internists and *n*=3 surgeons). For semi-structured telephone interviews and focus group discussions, guided questions were developed and discussed by the project group for content validity (see attachment 1 ). The focus group discussions were audio recorded or recorded by hand protocols; the telephone interviews were audiotaped and transcribed. According to the low interpretative purpose of our pilot study, we used a summative transcription by paraphrasing the keywords and statements of the interviews and the focus group protocol in accordance with Mayring [10], [11]. We defined categories in a deductive way based on the interview and focus group guideline and clustered the keywords and statements to these categories. AE, JJ, BH and KS did the summative transcription of the interviews or focus group discussions of their hospitals, and AE and KS clustered the summative transcription of the whole data set to the categories.

## 3. Results

According to the aim of our study, five categories were extracted from the material concerning the acceptance and feasibility of the provided EPA curriculum: (3.1.) The implementation process of the EPA curriculum and required resources, (3.2.) entrustment process, (3.3.) feedback sessions with supervisor, (3.4.) students’ and supervisors role perception and (3.5.) overall impact of EPAs on training conditions in the practical year. In the following sessions, the main aspects and challenges are described by opposing the students’ and supervising physicians’ statements and differentiating between faculty and student development. In table 2 (Tab. 2), we summarized the main critical factors in the implementation of the EPA curriculum.

### 3.1 The implementation process of EPA curriculum and required resources

To implement the EPA curriculum in the hospital wards, the project coordinators provided support – up to four hours per week and per ward or doctor’s office. This support included supervisor and student training as well as support in the daily ward routine. The aim of the offered support was to keep the project present in mind, to make suggestions for more student autonomy and to facilitate reflection on the final year’s ward training itself using targeted questions techniques. This close support during the implementation phase was offered to prevent returning to habitual behavior and was reduced bit by bit during the implementation period. Students and supervisors emphasized the necessity and helpfulness of such central assistance. The effort for the implementation of the EPA curriculum on the wards amounts to approximately 1 hour per week for the students and the supervisors.

In some areas, structural barriers were encountered during the implementation process. First, too few computer workstations and workplaces were available for students and there was inadequate access to and training for local clinical data processing programs. This clearly limited the possibilities for students to get necessary and appropriate information about their patients. Second, frequent turnover of supervisors and short training periods (on average, 8 weeks in a ward) of the students were detrimental to the entrustment process. Due to the recurring changes, the entrustment process started over and over again and the students had to prove themselves yet again to a new supervisor and therefore often did not get beyond a certain degree of responsibility. An approach to overcome this issue could be a structured and trustworthy handover between the supervisors, where the previous supervisor provides an appraisal of the student to the new physician. Some students and supervisors also reported that the documentation of the structured feedback sessions and work samples disrupted the daily clinical practice.

Concerning the required supervisors and wards, we planned to have a senior physician as well as an assistant physician. The ward physician is the person in charge of the student. Physicians in their first year of residency participated in this pilot study. It should be noted that these physicians are inexperienced and may still have difficulties solving certain tasks correctly and therefore cannot guide the students properly.

#### 3.2 Entrustment process

##### Student development

Many students reported that they experienced a stepwise process of assigning responsibility – starting with small and specific tasks that they had already performed many times before (e.g., venipuncture), followed by more complex tasks (e.g., history taking and physical examination) and finally conducting the defined complete EPAs. However, some students reported that the supervisors rarely supervised such activities directly and that they only assumed responsibility if they felt confident in performing the task. In other cases, the students reported that the implementation of EPAs made explicit what was already part of their training.

For the entrustment decisions, the supervisors reported self-assessment, motivation and dependability of the students as being crucial. The supervising physicians acknowledged that the direct supervision provided was still too little. Some supervisors felt insecure and uncomfortable with entrusting students with the responsibility of conducting ward rounds, prescribing medicine and documenting diagnoses and treatment plans because the students did not have specialized knowledge and prerequisites for those tasks. In General Practice rotation, supervisors emphasized the value of a stepwise explicit entrustment of tasks for the daily process.

##### Faculty development

Students and supervisors reported that supervision level 3 was regularly entrusted for the “inpatient admission” and “preparing medical consultation” EPAs. The “discharge of patient” EPA was entrusted only at one of the hospitals. During the discharge process, doctors provide important information to patients and outline further actions to be taken for the healing process. Therefore, the supervisors of the other three hospitals felt uncomfortable with entrusting this professional activity to the students even under indirect supervision. General practitioners felt most comfortable with entrusting the EPA “providing acute consultations and chronic patient care.” Students and supervisors reported difficulties concerning the implementation of the “general practical palliative care” EPA. The general practitioners reported that palliative care is a crucial area of General Practice. Entrusting this activity to the students would most likely lead to excessive demands on the patient. Concerning this issue, which is already in the development process, the highest achievable entrustment level for this EPA is Level 2. Nonetheless, in some cases, the implementation of this EPA was not possible.

Most supervisors hesitated to entrust professional activities under indirect supervision to their trainees. Thus, level 3c (student performs EPA independently under indirect supervision) was rarely achieved. Nonetheless, supervisors assigned responsibility more often for smaller and more specific tasks, such as “request of test results,” “patient’s medication history” or “history taking in general,” than for the more broad and complex EPAs. Finally, students mentioned that during the daily routine, supervisors often forgot that they had already entrusted certain tasks and EPAs to them and performed those themselves.

#### 3.3 Feedback sessions with the supervisor

Besides entrustment, structured observations, as well as appropriate and regular feedback, are central aspects of the EPA concept.

##### Student development

Students reported that they received feedback from their supervisors, but mostly in a spontaneous and unstructured manner; for instance, after inpatient admission, after writing physician’s letters or after ordering laboratory examinations. Students considered direct feedback following the activity at hand more helpful as it is more specific and effective than more general feedback during the monthly structured feedback sessions. The structured feedback sessions included more general topics such as students’ self-assessment, their experiences in the past weeks, their potential for improvement and the mutual expectations for future activities. Furthermore, students still felt that feedback sessions were often handled as a mandatory administrative act and not valued as an important teaching tool. Also, students mentioned that supervisors avoided negative feedback rather they provided a positive appraisal. However, the students appreciated the suggestions for improvement and consequently felt more confident in performing medical tasks.

The view of the supervisors was slightly different. In the context of this pilot project, they felt more legitimized to give not only constructive but also negative feedback. The supervisors felt well-prepared for the feedback sessions. Some saw the feedback as a reference for further improvement and a change in future behavior. The supervisors also emphasized that feedback sessions somehow improved the culture of communication. Some supervisors complained that student self-initiative seemed rare and was often completely missing, and stated that they saw the need for making an appointment for the feedback sessions clearly on the student side.

##### Faculty development

Relating to the organizational process of the feedback session, the first interview differed from the subsequent ones, as the supervisors were hardly able to judge the students' performance and abilities after such a short period. Therefore, student self-assessment played a central role. In general, the structured feedback sessions occurred in the afternoon with a duration of 10 to 30 minutes, frequently triggered by reminding coordinators, but they were also canceled often on short notice due to excessive workload.

#### 3.4 Students’ and supervisors role perception

##### Student development

Students reported that the EPA curriculum, in particular, changed their self-perception of their professional role. They felt more competent and empowered when working independently. The EPA curriculum especially supported their understanding of the diverse and wide range of professional medical tasks. Moreover, students felt more equally integrated into the medical team and regarded their experience as supportive of the team.

The supervisors observed attitude changes in the students as well. According to the supervisors, the students were more accountable for the patients they cared for, but in the presence of senior physicians, they perceived the students as much more reserved. However, some supervisors still expected more initiative and engagement from the students and criticized missing motivation as well as interest for the ward tasks or their medical specialty. However, they reported changing self-awareness in their role as student supervisors. They observed increasing attention to the training in the clinical electives due to the EPA curriculum. They felt that the EPA curriculum empowered students in a manner that consequently led to a reduction in clinical workload for the supervisors, allowing them to reinvest the gained time in more extensive teaching activities.

#### 3.5 The overall impact of EPAs on training conditions in the practical year

##### Faculty development

Overall, the concept was rated positively by the students and supervising physicians. Students assessed the concept as close to daily medical practice and supervisors stated that it helped them to structure the educational process. EPAs seem to support the clarification of the goals of the practical year to both the students and the supervisors.

The students emphasized that the EPA curriculum supported the training process in general during the practical year. The spectrum of the different EPAs – and especially their narrative description – gave them a concrete reference of the most important tasks which they would have to perform after their graduation. Furthermore, they felt the progression in competence development was somehow more explicit through the entrustment process, especially through explicit goal setting (“feed-forward”) during the feedback sessions. The written documentation of the training process helped them to explicitly identify deficits. The overall training process was perceived as more structured and less random compared to the usual training. In summary, students assumed that training with the EPA curriculum may improve their preparedness for residency.

## 4. Discussion

This study shows the first attempt to implement a structured EPA curriculum in the clinical electives during the final year of medical school (practical year) in Germany.

Telephone interviews and focus group discussions with students and supervisors show the first qualitative insight into the teaching process with the EPA curriculum during the practical year in internal medicine, surgery and general practice at four German medical schools. Overall, the students and the supervisors were very positive about working with EPAs and saw a promising perspective by introducing EPA already in the clinical electives at the end of undergraduate medical education. In summary, the analysis underlines three main aspects of implementing EPAs in undergraduate medical education:

### Learning goals of the EPA curriculum

The EPA curriculum gives a structured and intuitive guideline about the learning goals of the clinical electives and the training of clinical skills. Overarching goals of clinical electives are clearly stated and foster a better understanding and concreteness of needed clinical skills [12], [13]. On the one side, the students know what they can expect from their ward training, and on the other side, the supervisors also know which professional activities they can entrust to the students. The students also receive an overview of their future daily medical tasks at the beginning of their residency. However, we observed the challenge of handling the EPA curriculum in the context of high medical specialization, missing prerequisites on knowledge and skills for particular diseases and treatment plans. The students regret this missing knowledge and they saw it as an obstacle to assume particular tasks. The pilot study took place in the university hospitals with maximum care offer, though a large number of students will conduct their clinical electives in suburban hospitals with lower care offers. In these hospitals, the entrustment process could be implemented in a more sufficient way.

#### The granular structure of the EPA

The broader granularity of the EPA is perceived overall as suitable in this learning context. However, the implementation of the broader EPA seems difficult. Often, the supervisors assigned smaller and finer granular tasks, such as history taking, writing discharge letters or cannulating a patient, to the students. Nonetheless, it seemed that due to the broader definition of the EPA, the students and the supervisors got an important and initial explicit overview of the main clinical tasks and responsibilities, which they were supposed to account for by the end of medical school. The granularity of the EPA was a crucial aspect in discussions during the development process of this EPA curriculum and is also an ongoing critical discourse in the international community. Chen et al. stated that at the beginning of the training, the EPAs should represent smaller tasks, and the more experienced a trainee is, the broader the EPAs can be [14]. However, it was also stated that sometimes, an ad hoc entrustment for a full EPA, such as inpatient admission, as described by Lomis et al. [15], was possible for simple treatments. Diagnosing or treating more complex diseases were seldom entrusted due to supervisor or student uncertainty. Rather, a summative entrustment took place between the supervisors and the trainees in the feedback sessions.

#### Feedback

The feedback sessions in an explicit and structured manner are perceived as helpful and valuable for the students and the supervisors. Feedback is supportive of the training process and probably represents the most crucial element of the whole concept. The external feedback of the supervisors facilitates the students’ self-monitoring abilities and goal setting might also foster the learning process [16], [17]. However, the organizational aspect of the feedback sessions is seen as a major challenge. On the one hand, students often showed insufficient engagement to schedule the feedback sessions, and on the other hand, a huge workload on the wards was listed as an obstacle for running the feedback sessions. Furthermore, supervisors might often avoid naming students’ malpractice and therefore, the students would not have the opportunity to improve their professional skills and abilities. We could trace this phenomenon to a missing error culture at the wards. Due to the inability to handle mistakes, supervisors may also lack the skills to name these mistakes. Thus, students receive insufficient feedback.

The implementation of EPAs in the practical year necessitates a significant change of attitude toward learning and teaching in clinical electives which is inevitable for both the supervisors and the medical students. Supervisors expect a certain level of student motivation and engagement to be able to entrust them with professional tasks. Sterkenburg et al. [18] stated that, among other things, appraised experience, presented self-confidence and students’ requests are determining factors for entrusting professional activities. This study confirms that such aspects are important in order to entrust these tasks to medical students.

Moreover, some pilot study limitations should be considered when interpreting the results of this study. The participating supervisors were “handpicked” according to their superior engagement as medical teachers. Also, we assumed a high motivation of the participating students in the telephone interviews and focus group discussions. We tried to compensate for the small sample size by engaging four university hospitals and six general practitioners. The focus group discussions and telephone interviews were conducted by the project coordinators, who were predominantly faculty members; therefore, social desirability in the answers could not be precluded.

In the next steps, this EPA curriculum has to be tested in a broader setting to include more wards and disciplines. Further research may investigate the role of feedback in the entrustment process as well as the student perspective in entrustment situations. The aspects of facilitating the assignment of responsibility to the students could be another research focus.

Our recommendations for the implementation of an EPA curriculum in the undergraduate medical program include sufficient training for students and supervisors and support by a coordinator.

An evidence-based curriculum for introducing EPAs in medical faculties is necessary and already in preparation by the MFT working group. Beyond the EPA format, the training should pay particular attention to self-reflection concerning trust and entrustment. A co-training of physicians and students appears to be purposeful for a better mutual understanding of both groups.

The coordinator should give feedback to the physicians about their teaching and feedback processes with the students. The coordinator should also remind the supervising physicians of the feedback session as well as the students. In addition, it should be determined in advance who will make these appointments – the supervisor or the student. The EPA should be able to adjust to the specific circumstances and conditions of the ward or medical domain.

## 5. Conclusion

This study piloted the implementation of a newly developed EPA concept in the practical year of undergraduate medical education in Germany. Students were enabled to master more complex, independent tasks and assume responsibility for professional activities. The support offered by project coordinators was crucial for the successful implementation of the EPA curriculum in the clinical core electives. The feedback sessions support the learning impact of the clinical electives in general and the EPA curriculum in particular. The concept fosters the competency-based undergraduate medical education.

## Ethic

The local ethics committee has given a positive vote on the project (approval number: 6173R).

## Acknowledgements

We thank all participating students and supervisors. Special thanks to Dr. Folker Schneller, Prof. Dr. Ralf Gertler and Dr. Alexander von Werder, who supported the implementation phase at the Klinikum rechts der Isar of the Technical University of Munich. Also, special thanks to Prof. Dr. Matthias Schneider, Prof. Dr. Matthias Schott, Prof. Dr. M. Roden who supported the implementation at Düsseldorf University Hospital.

## Competing interests

The authors declare that they have no competing interests. 

## Supplementary Material

Leading questions for interviews

## Figures and Tables

**Table 1:**
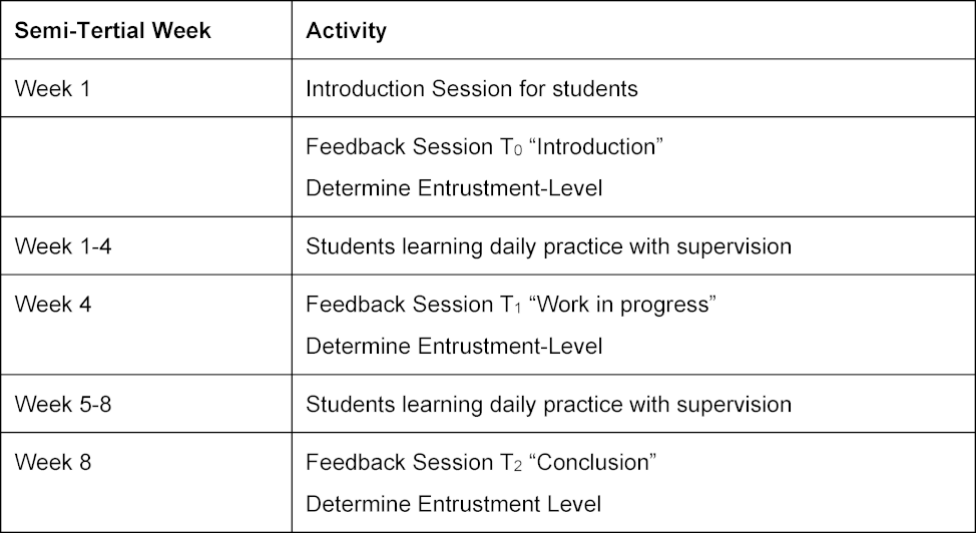
Schedule of pilot study example (eight weeks)

**Table 2:**
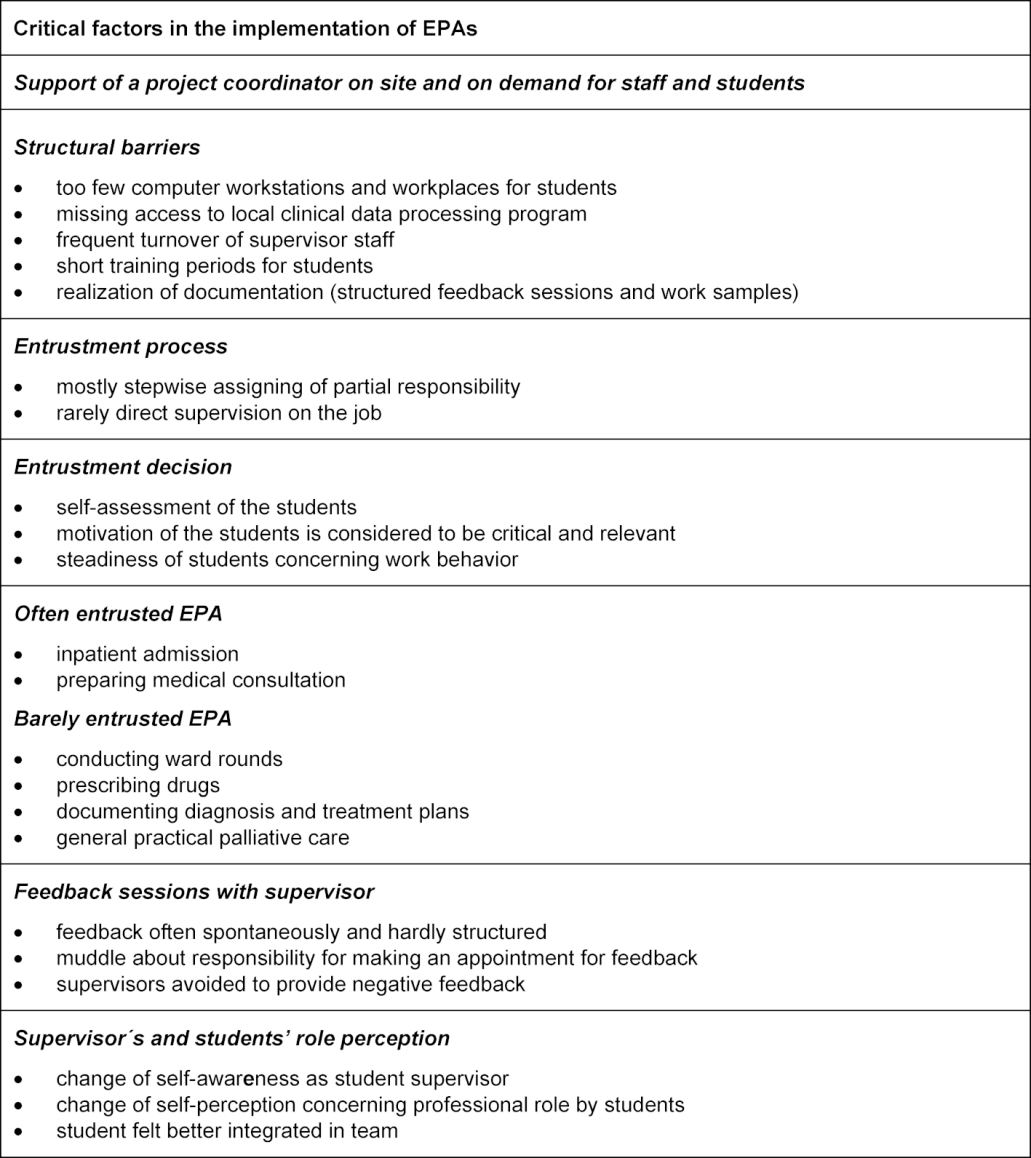
Critical factors in the implementation of EPAs
