# Supplementary material for: Implementing a logbook on entrustable professional activities in the final year of undergraduate medical education in Germany – a multicentric pilot study
Source: GMS J Med Educ. 2019 Nov 15;36(6):Doc69. doi: 10.3205/zma001277 (PMC6905372; doi:10.3205/zma001277)
Supplement: Leading questions for interviews [file JME-36-6-69-s-001.pdf]

Students	Teachers
In which situations has responsibility been assigned to you?<br>What kind of responsibility was assigned to you?<br>o From your perspective, what were the reasons for assigning responsibility to you?<br>o Did you feel confident in acting? Explain?<br>o How did the transfer of responsibility work?	How was the trusting process in clinical / everyday practice?<br>Key points to consider:<br>o Complexity of APT<br>o Carrying out punctual, regular observations, for example every 4 weeks<br>o Communication with the student including planning of the appointment feedback / observation dates by student<br>o Discussion in the interprofessional team regarding the assessment of the student
Did you perceive situations in which you are / were uncertain or afraid to assume responsibility?	How did you conduct the feedback interview?
What were the reasons for this uncertainty and anxiety?	How do you rate the provided examples for the observation of working results?
How did the trainers influence your training process?<br>o How did you react?	To what extent did the EPA concept change your approach to PJ students and the interprofessional exchange within the team?<br>o Can you give examples?
How did you receive feedback on your actions?	
How did the interviews with the instructor / supervisor take place? (Place, appointment, etc.)<br>o How did you feel about the talks?<br>o How did you handle the feedback from the instructor?<br>o Which aspects have significantly influenced your feedback reactions?	
At what intervals did these talks take place?<br>o How did the dates for these talks come about?<br>o How long did the talks last?	
How were your training experiences aligned with the EPA?	
Did you experience the EPA more as an opportunity or more as a duty?<br>o What is your experience based on?	
How did you prepare for EPA training?<br>o Do you feel sufficiently prepared? Please explain.	
To what extent has your perspective been changed by the EPA in the practical year?	
How do you assess the structural conditions for carrying out the tasks defined in the APT?<br>o (Computer workstation, access to patient data management systems etc.)	

Soon you will be certified and start your first day of work as an intern in a ward or in an outpatient clinic.<br>o In your opinion, how should the first weeks of your postgraduate training run?<br>o Do you have any wishes for changing compared to your training in the practical year?	
